# Explaining the impact of a women's group led community mobilisation intervention on maternal and newborn health outcomes: the Ekjut trial process evaluation

**DOI:** 10.1186/1472-698X-10-25

**Published:** 2010-10-22

**Authors:** Suchitra Rath, Nirmala Nair, Prasanta K Tripathy, Sarah Barnett, Shibanand Rath, Rajendra Mahapatra, Rajkumar Gope, Aparna Bajpai, Rajesh Sinha, Anthony Costello, Audrey Prost

**Affiliations:** 1 Ekjut, Ward No-17, Plot 556B, Potka, Po-Chakradharpur, Dist. West Singhbhum. Jharkhand Pin- 833102, India; 2 UCL Centre for International Health and Development, Institute of Child Health, University College London, 30 Guilford Street, London WC1N 1EH, UK

## Abstract

**Background:**

Few large and rigorous evaluations of participatory interventions systematically describe their context and implementation, or attempt to explain the mechanisms behind their impact. This study reports process evaluation data from the Ekjut cluster-randomised controlled trial of a participatory learning and action cycle with women's groups to improve maternal and newborn health outcomes in Jharkhand and Orissa, eastern India (2005-2008). The study demonstrated a 45% reduction in neonatal mortality in the last two years of the intervention, largely driven by improvements in safe practices for home deliveries.

**Methods:**

A participatory learning and action cycle with 244 women's groups was implemented in 18 intervention clusters covering an estimated population of 114 141. We describe the context, content, and implementation of this intervention, identify potential mechanisms behind its impact, and report challenges experienced in the field. Methods included a review of intervention documents, qualitative structured discussions with group members and non-group members, meeting observations, as well as descriptive statistical analysis of data on meeting attendance, activities, and characteristics of group attendees.

**Results:**

Six broad, interrelated factors influenced the intervention's impact: (1) acceptability; (2) a participatory approach to the development of knowledge, skills and 'critical consciousness'; (3) community involvement beyond the groups; (4) a focus on marginalized communities; (5) the active recruitment of newly pregnant women into groups; (6) high population coverage. We hypothesize that these factors were responsible for the increase in safe delivery and care practices that led to the reduction in neonatal mortality demonstrated in the Ekjut trial.

**Conclusions:**

Participatory interventions with community groups can influence maternal and child health outcomes if key intervention characteristics are preserved and tailored to local contexts. Scaling-up such interventions requires (1) a detailed understanding of the way in which context affects the acceptability and delivery of the intervention; (2) planned but flexible replication of key content and implementation features; (3) strong support for participatory methods from implementing agencies.

## Background

Community participation in health is a cornerstone of the World Health Organization's past and current strategies to achieve health for all [1,2]. Advocates believe that community involvement can make health services more accessible and sustainable, and that enabling communities to explore the consequences of health behaviour can yield lasting improvements in health outcomes. Another, more radical, expectation is that participation can enable people to gain the skills, information, and experience to challenge the social, political and economic structures that limit their agency [3]. More than thirty years after Alma Ata, multilateral development institutions, states and civil society organizations alike have embraced community participation in health, but meanings given to it vary widely between programs and measurable successes in improving health outcomes are scarce [4]. This has resulted in increasing concerns about the legitimacy and effectiveness of participatory interventions, with critics contending that they are often ill-defined, co-opted by powerful development actors to disguise top down, 'business as usual' implementation of externally designed programs, or so context-specific that their replicability and scalability is doubtful [5,6].

Despite this ambivalent legacy, commitment to community participation in health is enduring. This is especially true in the field of maternal and child health, where programmes have recognized the importance of community involvement to improve both the supply and demand for appropriate health services [7]. In addition, recent evaluations of participatory interventions have shown an impact on the intractable problem of high neonatal mortality in developing countries: two recent randomised controlled trials have demonstrated mortality reductions in rural, underserved communities of Nepal, and in eastern India [8,9]. The Makwanpur trial tested a participatory intervention with women's groups and found a 30% reduction in neonatal mortality after three years. In eastern India, the Ekjut trial (2005-2008) evaluated the impact of a similar programme on birth outcomes in three bordering districts of Jharkhand and Orissa. The intervention led to a 45% reduction in neonatal mortality over the last two years of the study and a 57% reduction in moderate maternal depression in the third year. Other evaluations of participatory interventions with women's groups are underway or recently completed in Bangladesh, urban India and Malawi.

While randomised controlled trials are considered the most rigorous method to evaluate the impact of complex interventions, attention must be given to the contextual and process factors that affect the efficacy of such interventions in order to determine how results might be replicated in non-trial settings [10]. Community mobilisation interventions raise specific evaluation challenges because their development, implementation and success involve a range of actors, activities and processes, often over prolonged periods of time [11]. Process evaluation helps to understand these factors by examining the context and implementation of an intervention, the mechanisms through which it may affect outcomes, and the response of the intervention target population. In an earlier publication we presented data on the impact of Ekjut's women's group intervention on neonatal and maternal health outcomes. This study presents data on the processes that underpinned the programme's delivery and results. We identify features of the context, intervention, and implementation methods that may have contributed to the impact on health outcomes, and provide recommendations for scaling-up similar interventions. We focus on maternal and newborn health outcomes other than maternal depression, as this will be the focus of a separate publication.

## Methods

Data collection for the Ekjut trial process evaluation began in July 2005, at the start of the women's group intervention. Table 1 outlines the evaluation's objectives, research questions, data collection tools, and methods. The process evaluation had six key objectives: 1. to describe the intervention, in principle and in practice; 2. to describe the social context within which the intervention was delivered; 3. to understand how and why the intervention affected group members; 4. to understand how and why the intervention affected those who do not attend groups within the same community; 5. to develop hypotheses about the mechanisms by which the intervention had the effects it did; 6. to test these hypotheses. In this study we attend to objectives 1 to 5, as addressing objective 6 would require additional analysis beyond the scope of this article.

**Table 1:** Process evaluation objectives, indicators and data collection

OBJECTIVE	INDICATORS	DATA COLLECTION METHODS	DATA SOURCE
**Objective 1: to describe the intervention in theory and in practice**			
	Issues encountered during the intervention piloting phase	Document review	Registers
	Facilitators' characteristics	18 group discussions with community members	FGD notes
	Facilitator's recruitment and training	Review of training and recruitment documents	Interview notes and training documents
	Facilitators' perceptions of the intervention	9 group discussions with facilitators	FGD notes
	Group formation	Analysis of data collected by facilitators	Group formation form
	Group discontinuation	Group discussions with facilitators and WG coordinators	FGD notes, visit notes
	Socio-demographic characteristics of women's group members and attendees	Analysis of surveillance data & data collected by facilitators	Surveillance questionnaire, meeting attendance sheets
	Regularity and cancellations of meetings	Analysis of cancellation forms	Facilitators register, cancellation forms
	Identification and prioritization of problems	Document review	Facilitators' register, meeting reporting forms
	Members' views on the identification and prioritization of problems and strategies	Analysis of evaluation forms	Evaluation form
	Identification of strategies, barriers and prioritization of strategies	Document review	Facilitators register, meeting reporting forms
	Community meetings	Document review	Facilitators' register
	Attendees' perceptions on community meeting	Document review	Structured observation notes by coordinators, DMs and Intervention managers
	Implementing strategies and measuring progress	Analysis of forms	Registers, reporting forma and group-wise record book to measure monthly progress
	Members' views on the implementation of strategies	Analysis of evaluation forms	Evaluation form
	Methods and process for cluster level community meetings	Document review	Facilitators' register
	Attendees perceptions on cluster level community meeting	Document review	Structured observation notes by coordinators, DMs and Intervention managers
	Evaluation of group activities	Analysis of group support forms	Group support form
	Evaluation of the phases of intervention	Analysis of phase wise evaluation forms	Phase-wise evaluation forms
	Group members' perception of the intervention and the implementing organization	3 group discussions with group members	FGD notes
**Objective 2: to describe the social context in which the intervention was delivered**			
	Information on terrain, health service provision, other NGO activities.	Health services mapping, group information forms and Group discussions with facilitators	Health services mapping forms, FGD notes
	People, cultural practices and livelihoods	Analysis of surveillance data, Group discussions and notes from meetings in the women's group cycle	Surveillance tool, FGD and meeting notes
	Profile of clusters	Analysis of forms and FGD notes	Population census of India 2001, districts record and FGD notes
**Objective 3: to describe the impact of the intervention on women's group members**			
	Perception of facilitators regarding behaviour change among group members	3 FGD with facilitators	FGD notes, case studies
	Perception of group members regarding their own behaviour change	244 group discussions with members	FGD notes, case studies
**Objective 4: to describe the impact of the intervention on non-group members**			
	Perception of facilitators regarding behaviour change among non-group members	3 group discussions with facilitators	FGD notes, case studies
	Perception of group members regarding behaviour change among non-group members	244 group discussions with group members (1 per group)	FGD notes, case studies

A process evaluation manager (SR) collated information on the intervention in theory and as implemented in the field, the social context in which it was delivered and its impact on group members and non-members. Data collection tools were developed in Hindi and Oriya. These included attendance forms, facilitator register books and focus group topic guides. The tools were iteratively adapted throughout the intervention period. The process evaluation included both qualitative data such as case studies, direct observation of meetings and focus group discussions, and quantitative data such as women's group meeting attendance records and data from the trial's main monitoring and evaluation questionnaire for information on group membership status. We used data collected both routinely (e.g. attendance sheets, festival calendars) and at specific time points (e.g. focus group discussions at the end of the intervention process). Table 1 outlines the research questions, data sources and analysis methods for the qualitative components of the study. SR carried out the analysis of qualitative data (group discussions and observation notes) by collating notes in Hindi, English and Oriya and analyzing them using a thematic 'framework' approach [12]. The analysis involved five steps: (1) familiarisation with data by reviewing notes in order to list key and recurrent themes; (2) development of a thematic framework on the basis of the process evaluation protocol questions (as described in table 1) and emerging themes; (3) indexing or applying the framework to the data in textual form by annotating the transcripts and observation notes; (4) charting, i.e. rearranging the data according to the appropriate part of the thematic framework; and (5) mapping and interpretation using the chart to define concepts and find associations between themes. While steps 1 to 3 was carried mainly by SR, most of the study authors took part in steps 4 and 5, contributing experiences and ideas to the final list of themes.

Group discussions and observations were preferred to other data collection methods because the majority of topics addressed were not sensitive and could be safely discussed within women's groups. In addition, these methods minimised disruptions to the intervention and capitalised on routine data collection. Respondents (group facilitators, members, other community members and stakeholders) were purposefully sampled for their insights into specific intervention components or processes and recruited by SR. Purposefully sampled participants included all group facilitators and women's group members who took part in the final focus group discussions, as well as community members and stakeholders who attended community meetings held as part of the intervention cycle. Verbal consent was sought from groups and community members prior to discussions, and community consent was obtained for the trial. NN analysed the quantitative data using SPSS (version 13). SR analysed the qualitative data thematically in local languages and discussed the results with the senior Ekjut team and AP for consolidation and inputs. The list of mechanisms reported in the results section was compiled by SR and AP with input from all authors. All but three of the authors (SB, AP and AC) were part of the implementation team. All names included in quotes and case studies are pseudonyms.

## Results

### The context

**Figure 1:**
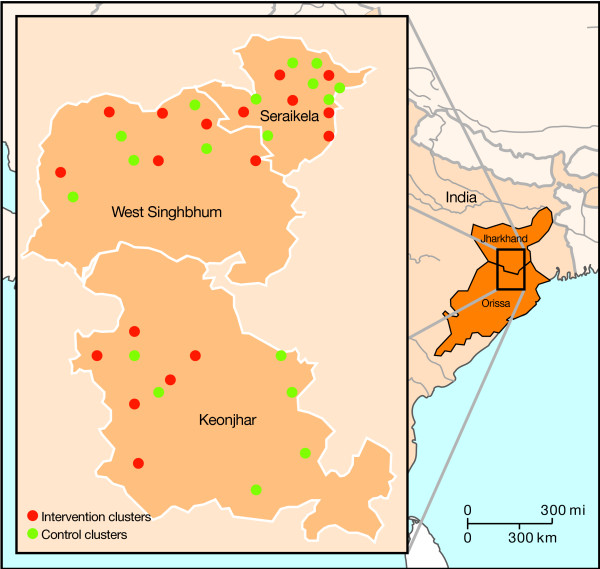
**Map of districts and clusters in the Ekjut randomised controlled trial**.

Documenting the context in which an intervention is developed and implemented is key to understanding its impact [13]. The Ekjut participatory learning and action cycle was carried out in 244 groups over three years in eighteen clusters within three bordering districts of Jharkhand (West Singhbhum and Saraikela Kharsawan) and Orissa (Keonjhar) (figure 1). The intervention areas were rural, largely tribal, and covered a population of 114 141, including 193 villages and 254 hamlets. Several tribal or *adivasi *(indigenous) groups inhabit these areas, including Ho, Santhal, Juang, Bhuiyan, Oraon and Munda communities. In both Jharkhand and Orissa, *adivasi *groups have distinct identities and strive to safeguard their social institutions and ancestral territories. Subsistence farming and foraging for forest produce are the main sources of livelihood, but these are being increasingly supplemented by wage labour, with men migrating to brick kilns or mines.

In 12 of the 18 Ekjut intervention clusters, villages were remote and located in hilly terrain surrounded by forests. Because of this physical isolation, villagers had limited access to health services. Among this largely tribal population, newborn health outcomes were poor: in the study clusters where 75% of the population belonged to Scheduled Tribes (ST), the neonatal mortality rate during the trial baseline period (2004-2005) was 58 per 1000 live births, and more than 80% of women delivered their newborns at home without skilled attendance. The intervention clusters were slightly disadvantaged compared with control clusters, with less access to primary health centres and fewer community health workers such as Anganwadi workers or Auxiliary Nurse Midwives. Traditional birth attendants carried out around 36% of home deliveries in the study clusters, and another 37% were carried out by relatives of pregnant women. Most *adivasi *communities in the study area were nature worshippers and interacted ritually with supernatural beings believed to reside in the home and natural environment. Health problems and illnesses were thus often attributed to supernatural causes and local diviners or private providers were commonly used to deal with problems in pregnancy and newborn illnesses.

The Indian government's flagship National Rural Health Mission (NRHM) programme was implemented in Jharkhand and Orissa during the study period (2005-2008). The NRHM seeks to improve access to quality health care in rural areas of India. In addition to health service strengthening, the NRHM supports a new community-based volunteer cadre, the Accredited Social Health Activist (ASHA) and seeks to strengthen Village Health Committees (VHCs) to address local health issues and monitor health services. The NRHM also promotes institutional deliveries through a voucher scheme (the Janani Suraksha Yojana or JSY) [14]. JSY was implemented during the Ekjut trial period but coverage varied greatly between states and districts, with a slow uptake in underserved areas. Although VHCs were being formed and ASHAs recruited, few ASHAs had been trained or deployed in the trial areas by the end of the study in July 2008. Table 2 shows the number of community health workers and health facilities in the study areas. Although primary health centers and community health centers were located in each of the clusters, villagers experienced multiple barriers to access, including physical distance, poor transport availability, and discrimination. Several NGOs operated in the study clusters but none carried out maternal and child health-related activities. In both intervention and control clusters there were pre-existing women's groups involved in credit and savings activities.

**Table 2:** Access to health services in the intervention and control areas

Community health workers and health facilities	Intervention areas	Control areas
Villages (n)	193	185
Anganwadi (Integrated Child Development Services) Centres	159	160
Auxiliary Nurse Midwives (ANMs)	63	59
Primary Health Centres	15	16
Community Health Centres	5	6
Sub--district Hospitals	1	1
District Hospitals	3	3
Villages with Sub-centres within 3 km	130	149
Villages with Primary Health Centres within 10 km	34	97
Villages with District Hospital within 30 km	121	133

There are noteworthy similarities between the Makwanpur and Ekjut trial sites: both are rural areas with poor access to health services. In both sites, over 80% of births occurred at home, and a high proportion of these home deliveries were assisted by relatives or traditional birth attendants. Despite these similarities, the context in which the Ekjut programme was delivered and the intervention itself also had distinctive features that may have contributed to the impact. These are discussed below.

### The intervention, its implementation and potential mechanisms

The intervention evaluated in the Ekjut trial was a participatory learning and action cycle of 20 meetings adapted from two previous experiences, the Warmi Project in Bolivia, and the Makwanpur women's group cycle in Nepal [15,16]. While the structure of the cycle was adapted from these two earlier studies, materials for individual meetings such as participatory games and strategies included the Ekjut team's own innovations as well as materials from two other women's groups interventions in Nepal and Malawi. During the trial period, 244 women's groups met monthly within groups of 15-20 to discuss problems related to pregnancy, childbirth, and the post-natal period; they were led by local facilitators trained in participatory communication methods who were not health educators but received basic training to discuss health problems during pregnancy and childbirth. The facilitator's average income for conducting a village meeting was 200 Indian Rupees, which is equivalent to the incentive that Accredited Social Health Activists receive for conducting community meetings. The cycle emphasised collective problem solving and planning and was divided into four phases (table 3). Group members organised community meetings at specific times during the cycle to share their learning with the wider community and enlist their support in implementing strategies to address problems in pregnancy and childbirth. Although most Ekjut groups met monthly, the entire intervention cycle lasted three years rather than the planned 22 months because of cancellations. These occurred mainly during festival, harvesting, and migration periods. 71.5% of groups met monthly throughout the whole intervention cycle, but all groups completed the entire cycle. Although there were political disturbances in the form of local strikes (*bandh*s), these did not affect the intervention as facilitators were resident within the clusters and were able to continue running the meetings.

**Table 3:** Meetings in the Ekjut participatory learning and action cycle

	AIM
PHASE 1	IDENTIFY AND PRIORITISE PROBLEMS
MEETING 1	Introduce the project and the women's group cycle
MEETING 2	Explore local practices and beliefs linked to pregnancy, childbirth and motherhood
MEETING 3	Identify maternal problems in the community
MEETING 4	Identify newborn health problems
MEETING 5	Prioritise the maternal and newborn problems the group wants to focus on
PHASE 2	PLAN STRATEGIES
MEETING 6	Discuss causes and solutions to local maternal and newborn health problems
MEETING 7	Identify strategies arising out of the solutions and understand opportunities and barriers before prioritizing them
MEETING 8	Discuss the process of sharing information on problems and strategies with the community
MEETING 9	Prepare a community meeting
	COMMUNITY MEETING
PHASE 3	IMPLEMENT STRATEGIES
MEETING 10	Discuss the implementation of strategies
MEETING 11	Review the progress of strategy implementation
MEETING 12	Discuss how maternal problems can be prevented
MEETING 13	Discuss how newborn problems can be prevented
MEETING 14	Discuss what home care solutions for selected problems
MEETING 15	Discuss facility-based care for selected problems
MEETING 16	Identify which problems are emergencies, prepare the group for emergencies and discuss ways of addressing delays in care-seeking
MEETING 17	Identify emergency and non-emergency problems, appropriate responses and referrals
MEETING 18	Learn about the activities of other groups and prepare for a cluster-level community meeting
	CLUSTER-LEVEL COMMUNITY MEETING
PHASE 4	ASSESS IMPACT
MEETING 19	Review the cluster community meeting, discuss the activities and achievements of the group and evaluate each phase of the cycle
MEETING 20	Discuss possible behavioural changes linked to the intervention that occurred in the wider community

Like the Makwanpur study, the Ekjut trial showed a substantial impact on neonatal mortality. This was largely mediated by improvements in safe delivery practices (hand washing, clean cord care and the use of safe delivery kits) for home deliveries rather than an increase in health service use. The Ekjut cycle retained three key characteristics of the Warmi and Makwanpur interventions: (1) local acceptability; (2) a participatory approach to the development of knowledge, skills and 'critical consciousness'; (3) community involvement beyond the groups. The ways in which these were operationalised in the context of the Ekjut trial are described below.

#### Acceptability of the intervention

Three main factors enhanced the intervention's acceptability: the recruitment and training of local facilitators, the use of locally appropriate discussion materials in meetings, and flexibility in the timing and content of meetings. Local trained facilitators are the main intervention implementers and are critical to its success [17]. In order to select facilitators, focus group discussions were held with elders, opinion leaders, headmen and women in three randomly chosen intervention clusters to identify selection criteria. Preference was given to local, literate married women, preferably daughters-in-law from the selected villages who had supportive families and could travel independently to conduct meetings. Senior Ekjut team members collated names of eligible candidates from villagers following visits to all clusters and invited them for a subsequent interview. Twelve of the 18 facilitators were married and from scheduled tribes, and most (11), had secondary education. Thirteen facilitators had past experience of group work through involvement with micro-credit and livelihood activities, but only two had done health related work. The facilitators' residential training was held in two phases with the first session lasting 5 days and the second session 2 days after a period of 6 months. The first training module emphasised listening and communication skills, and the first five meetings (identification and prioritizing of newborn and maternal problems) of the women's group cycle. In the second session, facilitators were trained in the process of developing stories depicting the causes and effects of health problems, making pictorial presentations of the stories to find solutions and prioritizing strategies to address problems. The training used a wide variety of methods including participatory exercises, group discussions, role-plays, story making, picture making and story narration. Participants were encouraged to keep a learning diary throughout the training, noting key learning points, training methods and notes for further action. The training module was developed using several training books and guides with feedback from Ekjut members. Some of the activities in the meetings such as the "but why" game [18], 'assigning responsibilities for the implementation of strategies' and the idea of using picture cards were adapted from Makwanpur; and some from MaiMwana in Malawi. Facilitators received ongoing support with documentation, field related problems and health-related questions during weekly or fortnightly review meetings with coordinators and senior team members. Weaker facilitators were given more attention and twinned with peers to increase their confidence. Facilitators earned the community's trust by being from the study area, respecting local practices, and knowing local languages. Perceptions of the facilitators' role are illustrated below, which presents qualitative data collected during the cycle's evaluation (phase 4). The following quotes were chosen by the research team during the qualitative analysis to illustrate perceptions of the facilitators' role:

*As I am from the same community it is easier for me to interact with the group and understand their health situation. Knowing the local language makes communication easier*. (Facilitator, Keonjhar, Phase 3 FGD)

*She is from our community, she is a friend, she helps us in solving our problems and makes us aware of the problems we suffer from by using picture cards and games, we consider her as a part of us and trust her*. (Group member, West Singhbhum, Phase 3 FGD).

*Group members believe our words and the contents discussed during the meetings. They implement them and when they get the benefits their trust strengthens*. (Facilitator, Saraikela Kharsawan, Phase 4 FGD)

Facilitators felt that the production and iterative adaptation of locally appropriate picture cards, stories, and participatory games increased acceptability and catalysed learning and planning within the groups. During the pilot phase, innovative facilitation methods were tried out and suitable techniques selected so that each women's group meeting had new activities, was participatory, and took less than two hours.

#### A participatory approach to developing knowledge, skills and critical consciousness

The implementation team and group members suggested that the structured, phase-wise content of the meeting cycle and its emphasis on collective problem solving contributed to learning and confidence building. This appears to have been a key determinant of the intervention's efficacy and acceptability. The following quotes from group members illustrate this:

We could not do much as individuals but as a group we could find a way to solve each other's problems. (Keonjhar, meeting 3)

Through story telling we could know some harmful practices and realised that because of some of the age-old practices many mothers and newborns might have lost their lives. (West Singhbhum, meeting 6)

It was easy to understand the causes and effects of maternal and newborn problems through picture card stories. (Saraikela Kharsawan, meeting 6)

By discussing the prevention and home care cards we realised that many diseases could be prevented. (Phase 4 FGD)

Games and role play helped to know the step by step process of handling emergencies. (Phase 4 FGD)

By sharing experiences with members of other groups we can learn from each other about the strategies that have benefited them. (Phase 4 FGD)

Review of the implemented strategies in each meeting helped us in performing our responsibilities properly. (Phase 4 FGD)

We are proud that to some extent we have helped in changing the behaviour of our group members and others who do not attend the meetings. (Phase 4 FGD)

**Figure 2:**
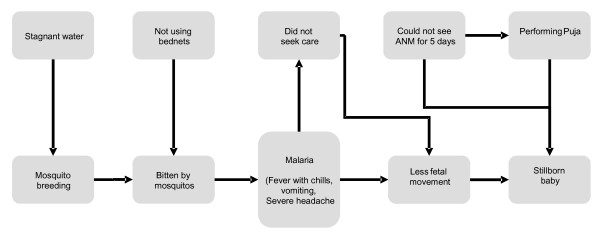
**Diagram used to create a story about the consequences of malaria in pregnancy**.

The implementation team (including facilitators) sought to make cause and effect linkages for health problems apparent through stories and problem-solving games. The process of creating stories was participatory and functioned as a training mechanism: while external staff learned about local practices, local facilitators familiarized themselves with preventive strategies for common problems and rehearsed the discussions which they would then carry out in the groups. Figure 2 shows an example of a story 'map' created by facilitators and Ekjut staff during a training session, which resulted in the following story:

Janaki lived in a remote village. When she was pregnant, she decided to visit her relatives who lived on the outskirts of a nearby town. Their house was in a relatively crowded area, with open drains and stagnant water. This is the ideal breeding place for mosquitoes. Her relatives advised her to use mosquito nets because they knew that there were many cases of malaria in this town. However, she ignored their advice and did not use a mosquito net, although she burnt a useful medicinal plant (neem) leaves. Soon afterwards she decided to return to her village but after a week she started to have fever with chills, severe headache and vomiting. She didn't seek care. When she noticed that the baby's movements had reduced she thought about seeing the Auxiliary Nurse Midwife, but the next health day was 5 days away, so instead she listened to the advice of her relatives, who advised her to do rituals and sacrifice two chickens and one goat. Soon afterwards she delivered a stillborn child.

Several stories focused on causes of maternal deaths, placing specific emphasis on the need for antenatal care and the prevention of delays in care-seeking. Although the Ekjut trial was not powered to detect a reduction in maternal mortality, we observed fewer maternal deaths in the intervention clusters: there were 49 maternal deaths over 3 years in the intervention clusters compared with 60 in control areas. Several stories such as the one included below, which sought to illustrate the causes of maternal deaths and stimulate discussions about prevention, may have played a part in this reduction:

Bamai got married at 16 and lived in a remote, hilly village. She became pregnant soon after and did not have any problems in early pregnancy. During one of her occasional visits to the village, the Auxiliary Nurse Midwife gave her a shot of Tetanus Toxoid and a few iron tablets. This was the only time in her entire pregnancy that Bamai met a health worker. The night she had labour pains, the *dai* (TBA) was called and said "pain has just started and this is her first pregnancy so it will take long, so call me when the pain increases". Throughout the night and the next day, Bamai's pain continued, and by the evening, when she had still not progressed, the relatives called the *dai*. The *dai* asked them to wait longer because she was attending to another delivery. Her mother-in-law noticed that the baby's fingers were visible through the vagina and that Bamai was exhausted as she had had nothing to eat or drink. Seeing her deteriorating condition, her relatives called in the ojha (local diviner) to perform some rituals and then sent for the village doctor. The village doctor came and gave her a hot injection, saying that she would deliver soon. But Bamai was tired and crying with pain. She requested her husband to take her to the hospital, so they started making arrangements to go to the nearest private hospital, which was 15 kms away. This took them a few hours: the roads were bad and they had to borrow money by mortgaging their land. Thirty hours after her pain had started Bamai reached the hospital unconscious and the attending nurse noticed that she was in shock and had lost a lot of blood. The nurse said that Bamai would need an operation for which blood was required because the baby was dead, the hand was lying outside, and the uterus had ruptured. There was no blood bank so the relatives started making arrangements for blood, but in the meantime Bamai was gasping and died.

Group members disseminated stories about pregnancy and delivery during community meetings on at least four occasions during the cycle. During the 3-year study period, facilitators and members narrated an estimated 976 new stories. This enabled women and the wider community to discuss cause and effect linkages, but also some of the more distal causes of health problems. This is important since one of the theoretical premises of the intervention is that behaviour change will occur if communities are able to analyse the cause and effect linkages of health problems, and then define ways in which they can influence these linkages. In the final stages of this process, communities would ideally understand both upstream and downstream determinants of health, identify the political and economic roots of ill health, and challenge actors responsible for perpetuating these. Writers such as Freire described this as the development of 'critical consciousness', or the process through which individuals and groups become conscious of the oppressive systems and actors that maintain some in poverty and ill health [19] We suggest that community mobilisation may have begun to catalyze critical consciousness among group members and the wider community, as evidenced in group members' support to local village health committees and their involvement of community health workers in discussions about entitlements to health services. However the development of a strong and sustainable community mobilisation movement is clearly a complex process that requires time and effort from both the community and the intervention implementers, and the effect of this may only be seen after some time.

#### From community involvement to community capacity

**Figure 3:**
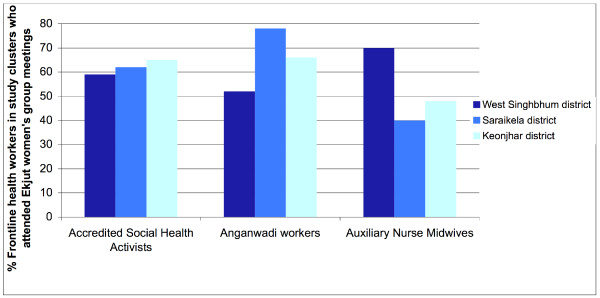
**Participation of frontline government staff at meetings in 3 districts**.

Two additional features of the intervention built on the participatory principles inherent to the women's group intervention but were unique to the Ekjut trial in their intensity and focus: the involvement of the wider community, including local community health workers, and the active targeting of marginalised groups and pregnant women. Group members garnered support for maternal and newborn health issues beyond the groups by actively involving the wider community in discussing their problems and strategies. This was done in three main ways. First, most of the groups were initially closed because they dealt with micro-credit activities, but, with the addition of the participatory cycle, groups became open to all community members and men, relatives of pregnant women and frontline government workers were free to attend. Second, members shared their problems and strategies with the wider community during village and cluster-level meetings. Third, community members, including men, offered support in the implementation of the groups' strategies. At the last meeting, an estimated 70% of group attendees were married women of reproductive age, 7% were men, and 23% were adolescent girls or unmarried women. Figure 3 shows the participation of frontline government staff in meetings in the study's 3 districts: ASHAs and Anganwadi workers were present in over 60% of meetings, and auxiliary nurse midwives (ANMs) attended an estimated 50% of meetings.

The groups' inclusiveness meant that different community members and decision-makers present during deliveries were likely to have attended meetings and therefore have increased awareness of maternal and newborn health issues. We reviewed several case studies highlighting the impact of groups on different community members and the consequences of this for health outcomes, and chose the following as illustration:

Sonia Munda suffered from swollen face and legs during her 8^th ^month of pregnancy. Her husband, who had attended some of the women's group meetings held in their village, asked her to go for a checkup because he knew it was a serious problem. When she refused, saying that it would be fine after she delivered, he asked a women's group member to convince her to go for treatment and they pressurized her to go for check-up during the ANM's visit. At the time of her delivery, she used the homemade delivery kit she had prepared. She delivered twins and did not bathe her babies, but instead wiped and wrapped them. After a few days, when she saw that another woman in the village also suffered from swollen face and legs, she accompanied her to the nearby Primary Health Centre. At the time of her delivery the group members took the initiative to convince her husband to have an institutional delivery and provided monetary support for taking her to the primary health centre, where she delivered normally.

The Ekjut trial surveillance data showed that over 37% of home deliveries in the study area were conducted by family members, including mothers-in-law, husbands, relatives and neighbours, so it is likely that group members or their relatives managed deliveries using information and skills from the meetings. Group members themselves became active health advocates in the community. In our surveillance data, members recalled making home visits, arranging transports for emergencies, providing financial help from the group's emergency fund (even to those who were not part of their savings groups), and counselling relatives of pregnant women. In total, members recalled providing assistance to 3822 pregnant women during the study period.

Women's group members described a progressive increase in community mobilisation to deal with health problems. They were able to make Auxiliary Nurse Midwives and Anganwadi Workers community health workers more accountable as these workers attended the meetings. The groups and their activities may also have catalysed community mobilisation and capacity beyond the health domain. The following quotes were selected during data analysis to illustrate this process:

*As for my knowledge, the people who are attending the meetings and discussing many new things about the health of mothers and newborns are explaining what they have learnt to five more people, as a result of which each and every person should know. These meetings are really helpful as we are only involved in trying to solve the health problems of the community through the help of community members. We believe that together we can bring about change*. (Group member and chairman of village education committee, community feedback meeting, Keonjhar)

*We used to live on our own, only concerned about our family well being, and others also used to only see their own interest. But now, as we are sharing and discussing our issues, we have developed a sense of bonding with each other and are helping each other in times of need*. (Group member, Phase 4 FGD)

#### Targeting vulnerable groups and pregnant women

In addition to its acceptability, its participatory approach to the development of knowledge, skills and 'critical consciousness', and the emphasis on community involvement beyond the groups, the Ekjut intervention had three further unique characteristics. First, the intervention team actively sought to work with the poorest communities in the study areas. Ekjut elected to work with existing PRADAN groups, an NGO primarily serving marginalised tribal areas. Ekjut also explicitly targeted areas that were predominantly inhabited by tribal people who had no or little land holdings, low literacy rates, and with many living below the poverty line. While 28 and 22% of the population in Jharkhand and Orissa are tribal, over 70% of women present in the first and last meeting of the Ekjut learning action cycle were tribal people. Ekjut also set up new groups in hamlets, which have poorer access to community health workers and health services than villages.

Second, the intervention team, facilitators, and group members invited pregnant women to join groups. Indeed, we observed that the attendance of pregnant women at group meetings increased over the three-year study period. During the first year, 18% (n = 546) of the women who delivered a baby attended Ekjut groups. This number increased to 38% (n = 1287) in the second year and reached 55% (n = 1718) in the third year.

Finally, the intervention had relatively high population coverage. In the Ekjut trial, the coverage of women's groups was 1 group per 468 population, compared with 1:756 in the Makwanpur trial and 1:1414 in a similar trial in Bangladesh, which did not yield a reduction in newborn mortality. We might therefore hypothesise that the Ekjut intervention had a significant impact on neonatal mortality because the key intervention characteristics described above were operationalised with local adaptations, the intervention had an adequate population coverage and high enrolment of pregnant women, the neonatal mortality rate was relatively high and a high proportion of neonatal deaths occurred at home.

### Challenges

The intervention team and group members faced several challenges. The team initially experienced difficulties in building a rapport with marginalised tribal communities and dealing with expectations of financial gain. Facilitators contended with dominant group members and cancellations during festivals and cultivation periods. They also managed the presence of men during sensitive discussions as well as rare disruptions from non-group members. They also had to ensure participation even during internal conflicts within villages. Group members were sometimes constrained by in-laws and TBAs (*dai*s) in the implementation of strategies, as some felt the contents of meetings went against traditional beliefs and practices. Despite these challenges however, all 244 groups completed the intervention cycle and members' attendance was maintained at more than 70% throughout the cycle.

There were considerable improvements in home care practices in the intervention areas, but increases in care-seeking were slower. As marginalised groups, tribal communities and the poorest among them had difficulties in accessing services. The remoteness of villages, poor access to transport and bad road conditions compounded these communities' social isolation; ANMs made irregular visits to villages and mothers had difficulties accessing antenatal check-ups. Several members had bad experiences in health facilities or reported that these were not equipped to deal with emergencies and had inconvenient opening times. In addition, care-seeking was higher at baseline in the trial control areas compared with intervention areas. Although there was a year on year increase in the proportion of women who received antenatal care, had an institutional delivery and received a postnatal check-up in the intervention clusters, the rate of increase was not high enough to catch up with the control clusters where simultaneous improvements also took place.

**Figure 4:**
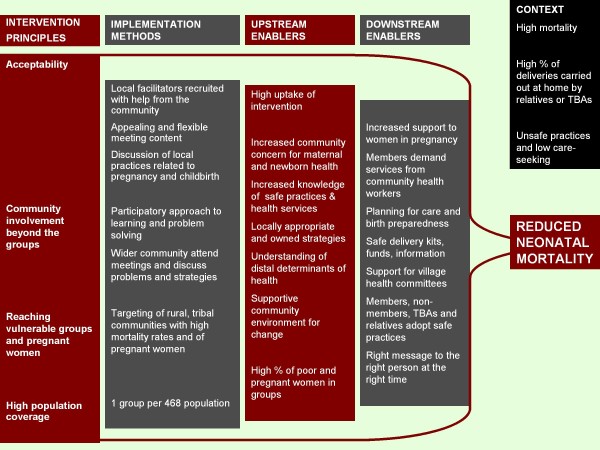
**Impact mechanisms of the Ekjut participatory intervention**.

Figure 4 summarises the key principles, characteristics and implementation methods that we believe contributed to the intervention's impact on neonatal mortality.

## Discussion

This process evaluation study had two key limitations: we relied on data collected and analysed by staff involved in the intervention's implementation and some of the intervention's shortcomings may have been under-reported. However, the active participation of some of the authors in the design and implementation of the intervention also gave them unique knowledge about its mechanisms and may therefore have been beneficial. Although the trial impact evaluation reported no significant changes in care-seeking between intervention and control areas, this may have been due to better access to services in control areas at baseline, and there is evidence that care-seeking started to improve in intervention clusters during the trial period, perhaps due to improved services under the NRHM and in particular to the expansion of the Janani Suraksha Yojana maternity incentive scheme.

The recently revised Medical Research Council (MRC) framework for the evaluation of complex interventions argues that "complex interventions may work best if tailored to local circumstances rather than being completely standardized" [20]. We hypothesize that this and other community mobilisation interventions can improve maternal and newborn health outcomes if its six key characteristics are carefully considered and operationalised by implementing agencies. These are: (1) acceptability; (2) a participatory approach to the development of knowledge and skills; (3) community involvement beyond the groups; (4) a focus on marginalized communities; (5) the active recruitment of newly pregnant women into groups; (6) high population coverage.

Community mobilisation challenges existing models of public health intervention delivery: mobilisation through groups is not a discrete intervention where impact is delivered linearly from implementers to recipients. Instead, implementers, facilitators, group members and community members are all in turn 'designers', 'implementers' and 'recipients' of learning and change. The recognition that all these participants can and must contribute is critical to trust, and thereby to behavior change. This cyclical learning process is different to methods used in traditional health education or even 'behaviour change communication' models, but must be understood and respected by implementing organizations in order to support community mobilisation in the face of multiple local and external challenges. The model also challenges existing community interventions to improve maternal and newborn health outcomes, many of which rely largely on home visits by community health workers and involve communities mainly through awareness raising meetings.

Can and should community mobilisation approaches be replicated and scaled-up to improve maternal and newborn health? We believe that they should, in combination with appropriate outreach and health services strengthening activities. The Ekjut and Makwanpur studies have shown that interventions building on community mobilisation are effective in high mortality settings where a high proportion of deaths occur in the community from causes such as sepsis and hypothermia. This impact may be more difficult to achieve in settings where further mortality reduction is largely dependent on improvements in health service access and quality, in particular emergency obstetric care, but where structural factors hinder communities' ability to act on these. The intervention can be a complement to other models, including home visits, which have impacted on mortality in studies such as the Shivgarh and Projahnmo trials [21,22]. For countries where maternal and newborn mortality rates remain unacceptably high and other interventions such as home visits have not yet reached adequate coverage, community mobilisation interventions such as the Ekjut PLA cycle can lead to substantial change [23].

## Conclusions

The Ekjut trial is an example of a successful participatory intervention that has shown a tangible impact on seemingly intractable health outcomes. Participatory community mobilisation interventions may influence maternal and child health outcomes if their key intervention principles are preserved and tailored to local contexts. Scaling-up this community mobilisation intervention will require a detailed understanding of the way in which changing contexts, delivery mechanisms, and implementation styles will affect key characteristics of the intervention. If combined and locally tailored, community mobilisation, improvements in health services, and the involvement of community health workers have the potential to yield lasting change for mothers and newborns.

## Competing interests

The authors declare that they have no competing interests.

## Authors' contributions

SR and AP wrote the first draft of the paper and coordinated all subsequent inputs. SR, NN, PT and AP contributed to the data analysis. SB, SR and NN designed the process evaluation protocol for the Ekjut trial. All authors contributed to the final analysis and recommendations.

## Pre-publication history

The pre-publication history for this paper can be accessed here:

http://www.biomedcentral.com/1472-698X/10/25/prepub

## References

[B1] World Health Organisation Declaration of Alma Ata 1978 International Conference on Primary Health Care, Alma Ata

[B2] Lawn JE Rohde J Rifkin S Were M Paul VK Chopra M Alma Ata thirty years on: revolutionary, relevant, and time to revitalise Lancet 2008 372 917 27 10.1016/S0140-6736(08)61402-6 18790315

[B3] Rifkin SB Ten best readings on community participation and health African Health Sciences 2001 1 43 47 PMC2704450 12789133

[B4] Morgan L Community participation in health: perpetual allure, persistent challenges Health Policy and Planning 2001 16 221 230 10.1093/heapol/16.3.221 11527862

[B5] Cornwall A Brock K What do buzzwords do for development? A critical look at 'participation', 'empowerment' and 'poverty reduction' Third World Quarterly 2005 26 1043 1060 10.1080/01436590500235603

[B6] Cooke B Kothari K Participation: The New Tyranny? 2005 London: Zed Books

[B7] Rosato M Laverack G Howard Grabman L Tripathy P Nair N Mwansambo C Azad K Morrison J Bhutta Z Perry H Rifkin SB Costello A Community participation: lessons from maternal newborn and child health Lancet 2008 372 962 971 10.1016/S0140-6736(08)61406-3 18790319

[B8] Manandhar D Osrin D Shrestha B Mesko N Morrison J Effect of a participatory intervention with women's groups on birth outcomes in Nepal: cluster randomized controlled trial Lancet 2004 364 970 979 10.1016/S0140-6736(04)17021-9 15364188

[B9] Tripathy PK Nair N Barnett S Mahapatra R Borghi J Rath S Rath S Gope R Mahto D Rashminayana R Patel V Prost A Costello AM Effect of a participatory intervention with women's groups on birth outcomes in Jharkhand and Orissa, India: the Ekjut trial Lancet 2010 375 1182 1192 10.1016/S0140-6736(09)62042-0 20207411

[B10] Oakley A Strange V Bonell C Allen E Stephenson J the RIPPLE study team Process evaluation in randomised controlled trials of complex interventions BMJ 2006 332 413 416 10.1136/bmj.332.7538.413 16484270 PMC1370978

[B11] Butterfoss FD Process evaluation for community participation Annual Review of Public Health 2006 2006 27 323 40 10.1146/annurev.publhealth.27.021405.102207 16533120

[B12] Pope C Ziebland S Mays N Qualitative analysis in healthcare research BMJ 2000 8 320 114 116 10.1136/bmj.320.7227.114 PMC1117368 10625273

[B13] Wight D Obasi A Stephenson J, Imrie J, Bonell C Unpacking the Black Box: the importance of process data to explain outcomes Effective Sexual Health Interventions: Issues in Experimental Evaluation 2003 Oxford: Oxford University Press 151 166 full_text

[B14] National Rural Health Mission Report of Common Review 2008 Mission: Jharkhand. Ranchi, December http://www.mohfw.nic.in/NRHM/CRM/Jharkhand_2nd_CRM_Report.pdf [Accessed 04/09/09]

[B15] Howard Grabman L De Koning K, Martin M Planning together: developing community plans to address priority maternal and neonatal health problems in rural Bolivia Participatory research in health issues and experiences 1996 London, Zed Books 153 163

[B16] Morrison J Tamang S Mesko N Osrin D Shrestha B Manandhar M Women's health groups to improve perinatal care in rural Nepal BMC Pregnancy and Childbirth 2005 5 6 10.1186/1471-2393-5-6 15771772 PMC1079874

[B17] Alcock GA Shah More N Patil S Porel M Vaidya L Community-based health programmes: role perceptions and experiences of female peer facilitators in Mumbai's urban slums 2009 Health Education Research 10.1093/her/cyp038 PMC2777946 19651641

[B18] Werner D Where there is no doctor: a village health care handbook 10.1136/bmj.317.7171.1532 PMC1114362 9831604

[B19] Freire P Education for Critical Consciousness 2005 New York: Continuum International Publishing Group

[B20] Craig P Dieppe P Macintyre S Michie S Nazareth I Petticrew M Developing and evaluating complex interventions: the new Medical Research Council guidance BMJ 2008 337 a1655 10.1136/bmj.a1655 18824488 PMC2769032

[B21] Kumar V Mohanty S Kumar A Misra RP Santosham M Awasthi S Baqui AH Singh P Singh V Ahuja RC Singh JV Malik GK Ahmed S Bhandari M Darmstadt GL Effect of community-based behaviour change management on neonatal mortality in Shivgarh, Uttar Pradesh, India: a cluster-randomised controlled trial Lancet 2008 372 1151 62 10.1016/S0140-6736(08)61483-X 18926277

[B22] Baqui AH El-Arifeen S Darmstadt GL Ahmed S Williams EK Seraji HR Mannan I Rahman SM Shah R Saha SK Syed U Winch PJ Lefevre A Santosham M Black RE Effect of community-based newborn-care intervention package implemented through two service-delivery strategies in Sylhet district, Bangladesh: a cluster-randomised controlled trial Lancet 2008 37 936 44 10.1016/S0140-6736(08)60835-1 18539225

[B23] Nair N Tripathy P Prost A Costello A Osrin D Community-based approaches to improve newborn survival in low-income countries PLoS Med 7 4 e1000246 10.1371/journal.pmed.1000246 20386728 PMC2850383

